# Microwave-assisted cassava pulp hydrolysis as food waste biorefinery for biodegradable polyhydroxybutyrate production

**DOI:** 10.3389/fbioe.2023.1131053

**Published:** 2023-03-06

**Authors:** Patiya Prasertsilp, Kobchai Pattaragulwanit, Beom Soo Kim, Suchada Chanprateep Napathorn

**Affiliations:** ^1^ Department of Microbiology, Faculty of Science, Chulalongkorn University, Bangkok, Thailand; ^2^ Department of Chemical Engineering, Chungbuk National University, Cheongju, Chungbuk, Republic of Korea; ^3^ International Center for Biotechnology, Osaka University, Osaka, Japan

**Keywords:** polyhydroxybutyrate, biorefinery, cassava starch industry, microwave, low-cost PHB production, valorization of agri-food wastes

## Abstract

Cassava pulp is one of the most abundant agricultural residues that can cause serious disposal problems. This study aimed to apply a biorefinery approach by examining the feasibility of microwave-assisted cassava pulp hydrolysis to attain sustainable management and efficient use of natural resources. Four factors, namely, the liquid-to-solid ratio (20 mL/g, 10 mL/g, 7.5 mL/g, and 5 mL/g), types of acids (H_2_SO_4_ and H_3_PO_4_), watt power (600 W, 700 W, and 800 W) and time (3, 5 and 8 min), were carefully investigated. The highest fermentable sugar content of 88.1 g/L ± 0.7 g/L (0.88 g fermentable sugars/g dry cassava pulp) was achieved when 20 mL/g cassava pulp was hydrolyzed with 2.5% (v/v) H_2_SO_4_ under microwave irradiation at 800 W for 8 min. Glucose was a major product (82.0 g/L ± 5.2 g/L). The inhibitor concentration was 5.17 g/L ± 0.01 g/L, and the levulinic acid concentration was 5.15 g/L ± 0.01 g/L. The results indicated that the liquid-to-solid ratio, diluted acid concentration, irradiation watt power and time were important factors in producing fermentable sugars from acid hydrolysis under microwave irradiation. The crude hydrolysate was used for PHB production by *Cupriavidus necator* strain A-04. The hydrolysate to nutrients ratio of 30:70 (v/v) yielded a cell dry weight of 7.5 g/L ± 0.1 g/L containing PHB content of 66.8% ± 0.3% (w/w), resulting in a yield $Y_{P/S}$ (g-PHB/g- $S_{PHB}$ ) of 0.35 g/g. This study demonstrated that the microwave-assisted cassava pulp hydrolysate developed in this study provided a high amount of glucose (88.1% conversion) and resulted in a low concentration of inhibitors without xylose; this was successfully achieved without pregelatinization, alkaline pretreatment or detoxification.

## Graphical Abstract

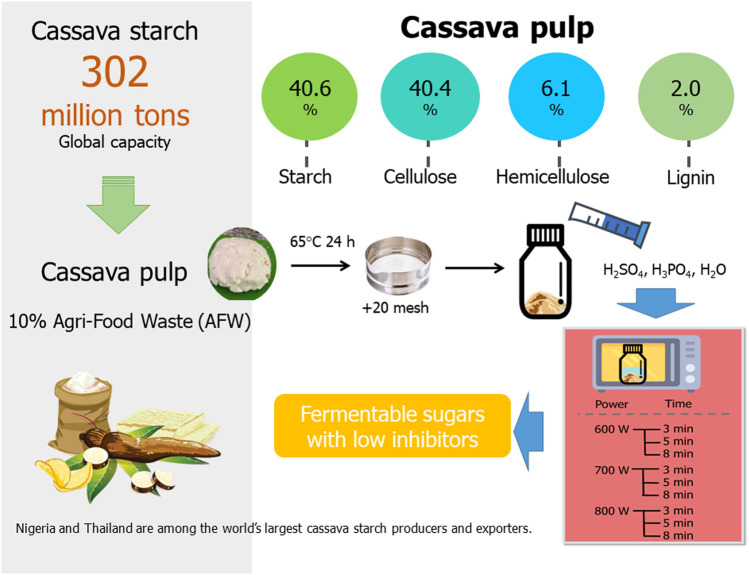

## 1 Introduction

Recently, biorefinery has been adopted as a bottom line technology in biotechnological processes because it integrates white and green biotechnology by converting lignocellulosic wastes into high-value-added products to attain sustainability (Barcelos et al., 2018; Areepak et al., 2022). Approximately 181.5 billion tons per year of lignocellulosic wastes have been significantly generated, whereas only 8.2 billion tons per year are used to produce value-added food and non-food bioproducts; however, 1.2 billion tons from agriculture residues are still leftover (Ubando et al., 2020; Areepak et al., 2022). According to the report of the Food and Agriculture Organization (FAO) in 2019, approximately 14 per cent of the global food, which is worth $400 billion annually, was lost after it was harvested before arriving at the stores (FAO, 2019). Meanwhile, the United Nations Environment Program (UNEP)’s Food Waste Index Report reveals that an additional 17 per cent of food is wasted in retail and by consumers, particularly in households (United Nations Environment Programme, 2021). Theoretically, individuals who are a part of the food supply chain are responsible for making their own decisions so that they will maximize their own profits or consumers’ benefits. For example, a food processor might experience some physical food loss that could be reduced by investing in better operational management or more advanced equipment; however, the cost of doing so would be greater than the potential value of the food that could be recovered, so the food processor decides not to proceed that tasks (Food and Agriculture Organization, 2019). Therefore, agricultural food waste evaluation is crucial from both an environmental and financial standpoint. The impact of agricultural food waste has a detrimental influence on food security and nutrition and notably contributes to greenhouse gas (GHG) emissions, serious environmental issues, natural ecosystem degradation, and biodiversity loss. There is an urgent need to significantly boost the usage of agricultural food wastes, which are renewable resources. Predominant examples of renewable resources include crop wastes and leftovers produced during crop production and food processing. These factors motivate scientists to devise strategies for turning agricultural and industrial food wastes into useful value-added products. One of the examples of agricultural food wastes is casava pulp derived from cassava industries. The negative impact of cassava wastes on the environment and health has been one of the challenges. In this regard, the organic wastes from cassava-based industries have enormous potential to generate diverse higher-value products by adopting a biorefinery concept (Pandey et al., 2000; Zhang et al., 2016). For instance, casava pulp can be used as the primary substrates for microorganisms to produce valuable products such as enzymes, antibiotics, polysaccharides, antioxidants, organic acids, biofuels, biogas, biosurfactant, biopolymers and other beneficial biochemical products (Zhang et al., 2016). It has a considerable potential for bioconversion into value-added products *via* biorefinery due to its complex biochemical compositions containing high organic content, which enables the cassava industries to be both economically and environmentally sustainable.

Cassava (*Manihot esculenta*), widely recognized as manioc, yucca, and tapioca plant, is the third largest food carbohydrate source cultivated in tropical and subtropical regions (Tur and Bibiloni, 2016). It has been widely used in i) human food, ii) animal feed, iii) biofuel, and iv) biotechnological factories (Sowcharoensuk, 2021). Approximately 302 million tons of cassava were produced worldwide in 2021/22. The biggest cassava starch producer is Nigeria (21.6%). Thailand has been ranked as the second largest cassava starch producer (10.7%) and exporter with a production capacity of 35.1 million tons (Office of Agricultural Economics, 2021). Consequently, cassava pulp is also generated in a large amount, approximately 10% of raw material, and contains 40%–60% of residual starch trapped in residual fibers (Sudha et al., 2015). Cassava pulp is easily available, inexpensive, plentiful, and renewable agriculture residues (Sriroth et al., 2000). Additionally, the utilization of cassava pulp is considered as the second-generation biorefineries since it is biomass waste and non-edible crops as feedstock (Mathioudakis et al., 2017). Despite the fact that a lot of cassava pulp is generated, the issue persists since the process is unmanaged and the cassava waste is typically kept in an open location. Accordingly, it immediately spoils and emits an unpleasant and strong scent. Moreover, the use of cassava pulp has been proposed in various applications, such as animal feeds (Diarra and Devi, 2015; Dagaew et al., 2022; Pongsub et al., 2022), bioethanol (Rattanachomsri et al., 2009; Siriwong et al., 2019), succinic acid (Sawisit et al., 2015), lactic acid (Thongchul et al., 2010; Gali et al., 2021), fertilizer (Phun-iam et al., 2018; Hasanudin et al., 2019), biogas (Lerdlattaporn et al., 2021), biodegradable polymer (Hierro-Iglesias et al., 2022) and biocomposites (Nguyen et al., 2020; Nithikarnjanatharn and Samsalee, 2022). Clearly, high value-added products can be created from leftover cassava wastes generated by the cassava industry. In addition to the direct use of cassava pulp, our focus was on the preparation of fermentable sugars for the microbial cultivation to produce value-added products because of its rich organic nature and low ash content owing to a great opportunity to utilize it in various biorefinery platforms (Pandey et al., 2000; Zhang et al., 2016).

This study focuses on development of fast and simple method for conversion of cassava pulp to fermentable sugars with low inhibitor concentrations. The hydrolysis of cassava pulp has been extensively studied through alkaline pretreatment of lignin (Kanchanasuta et al., 2020), acid hydrolysis (Binder and Raines, 2010), enzyme hydrolysis (Rattanachomsri et al., 2009; Bunterngsook et al., 2017) and a combination of the above methods (Thongchul et al., 2010). The significant time and energy consumption of the aforementioned technologies are their shortcomings. This generally happens when optimizing different material properties by adjusting existing strategies. More recently, some scientists have reported microwave-assisted hydrolysis based on a microwave synthesis reactor that offers temperature control, time and radiation watt input. Microwave-assisted hydrolysis has been of interest because it is a quick and simple method for converting biomass into useful products (Tsubaki et al., 2008; Yoshida et al., 2010; Zhang and Zhao, 2010; Sweygers et al., 2018; Shangdiar et al., 2022). These reports have inspired us to use household microwaves to assist diluted acid hydrolysis using cassava pulp for biodegradable polyhydroxybutyrate (PHB) production. The objective of this study was to develop direct hydrolysis and saccharification processes based on the synergistic action of diluted acid and microwaves for cassava pulp without a pregelatinization step. Crude hydrolysate without inhibitor removal obtained from optimized conditions was applied in PHB production using the levolinic acid-tolerant, glucose- and fructose-utilizing bacterium *Cupriavidus necator* strain A-04 (Sukruansuwan and Napathorn, 2018). This study proposes a non-pretreatment and non-hyperthermal saccharification process as an alternative method for bioconversion of cassava pulp to PHB and other value-added products.

## 2 Article types

Original Research Articles in a Research Topic Title: “The Value of Microbial Bioreactors to Meet Challenges in The Circular Bioeconomy".

Topic Editor(s): Wan Abd Al Qadr Imad Wan Mohtar, Neil J Rowan, Zul Ilham.

## 3 Materials and methods

### 3.1 PHB-producing strains

*Cupriavidus necator* strain A-04, which was previously reported to tolerate inhibitors in pineapple waste hydrolysate and to convert fermentable sugars without detoxification to PHB, was used in this study (Chanprateep and Kulpreecha, 2006; Chanprateep et al., 2008; Sukruansuwan and Napathorn, 2018; Wongmoon and Napathorn, 2022). The bacterial strain was cultured on a nutrient agar slant at 4°C. Stock cultures were maintained at −80°C in a 15% (v/v) glycerol solution.

### 3.2 Carbon sources

Fresh casava pulp was obtained from Thai Wah Public Company Limited (Thailand). It was dried in a hot-air oven (UN55, Memmert GmbH + Co. KG, Schwabach, Germany) setting the temperature at 65°C overnight and milled using a high-speed blender with maximum speed at 45,000 rpm (1,800-W, Healthy mix GP 3.5, Taiwan). Next, they were sieved with 20 mesh to obtain particle sizes less than 0.841 mm (−20 mesh). The moisture content was determined according to AOAC Official Method 934.06 (AOA, 1999). The chemical compositions of casava pulp were determined according to the Technical Association of Pulp and Paper Industry (TAPPI) standard methods parameters (TAPPI T 204 cm-07, TAPPI T203 om-09, TAPPI T9 m-54, TAPPI T222 om-15, TAPPI T-211). The remaining cassava starch was extracted from cassava pulp (Ahtong and Charoenkongthum, 2002).

### 3.3 Microwave irradiation

Microwave irradiation was conducted using a household microwave oven (max output; 800 W, ME711K model, Samsung Electronics Co., Ltd., Malaysia). The position of the hot spot inside the microwave chamber was identified using copying paper. The amount of dried and sieved cassava pulp was varied from 5, 10, 15 and 20 (w/v) and dissolved in 100 mL of 0.25% (v/v) H_2_SO_4_ or H_3_PO_4_ and microwaved in a closed system in a 500 mL Duran^®^ round bottle with a high-temperature red polybutylene terephthalate (PBT) screw cap with a polytetrafluoroethylene (PTFE) silicone cap liner. The types of acid, concentrations, watts power and irradiation time were investigated as one factor at a time. The concentrations of H_2_SO_4_ and H_3_PO_4_ were varied individually from 0%, 0.25%, 0.5%, 1%, and 2% (w/v) using a microwave with 800 W for 8 min by placing an Erlenmeyer flask on the identified hotspot. Then, the watt output was varied from 600 W, 700 W, and 800 W, and the heating time was varied from 3, 5 and 8 min. Water was used as a control experiment under identical conditions. The temperature of the resulting samples was measured. The supernatant was filtered through Whatman filter paper (No. 1, pore size of 11 μm, Sigma‒Aldrich Corp., St. Louis, MO, United States). The filtrate was neutralized using 2 M NaOH to obtain neutralized hydrolysate.

### 3.4 Culture conditions for PHB production from hydrolysate

Firstly, seed culture was prepared in 500-mL Erlenmeyer flasks containing 100 mL of preculture medium. The seed culture medium formula was 2 g/L yeast extract, 10 g/L polypeptone and 1 g/L MgSO_4_∙7H_2_O (Yabueng and Napathorn, 2018). The seed culture was grown on a rotary incubator shaker (Innova 4,300, New Brunswick Scientific Co., Inc., Edison, NJ, United States) at 30°C and 200 rpm for 24 h. The bacterial cells were separated from seed culture medium by centrifugation and washed to remove residual medium with 0.85% sodium chloride solution and resuspended in 100 mL 0.85% sodium chloride solution. Next, to promote the synthesis of PHB, the cells suspension were transferred into a production medium which was the same as previously described (Sukruansuwan and Napathorn, 2018) with modifications to favor cell growth and PHB production. The ratio of hydrolysate to production medium was varied from 100:0%, 90:10%, 80:20%, 70:30%, 60:40%, and 50:50% (v/v). The cultivation was performed in a shaken flask cultivation at 30°C and incubated on rotary shaker with shaking speed at 200 rpm for 96 h. Culture samples were harvested at 12-h intervals.

### 3.5 Analytical methods

Bacterial cell growth was monitored as cell dry mass, CDM, which was performed by cell filtration. Briefly, cellulose nitrate membrane with pore size of 0.22 μm (Sartorius, Goettingen, Germany) was weighted beforehand. Then, 5 mL of the culture broth was filtrated and dried at 80°C overnight and kept in desiccators until a constant weight was obtained. The residual cell mass, RCM, was calculated by subtracting the amount of PHB from CDM. The whole-cell methyl esterification was performed with additional modifications from Bruanegg et al. (1978) to quantify PHB in dried cells by gas chromatography (Model CP3800, Varian Inc., Walnut Creek, CA, United States) using a Carbowax-PEG capillary column (0.25-μm df, 0.25-mm ID, 60-m length, Varian Inc.) (Braunegg et al., 1978; Chanprateep et al., 2001). The benzoic acid was used as internal standard. The commercialized natural origin PHB was used as external standard (Sigma‒Aldrich Corp.). The concentration of monosaccharide (xylose, glucose, fructose, galactose and arabinose) and disaccharide (sucrose and cellobiose) in hydrolysate were analyzed by a high-performance liquid chromatograph (Model 626, Alltech Inc., Nicholasville, KY, United States) as described previously (Sukruansuwan and Napathorn, 2018). The eluent solution was water at a flow rate of 0.6 mL/min. The operating temperature was set at 60°C. The concentration of inhibitors (levulinic acid, 5-hydroxymethyl furfural (5-HMF) and furfural) in hydrolysate were analyzed using HPLC equipped with an ultraviolet (UV) detector setting a wavelength of 285 nm (Prostar 335, Varian Inc., Walnut Creek, CA, United States) and a ChromSpher C18 column (4.6-mm ID × 250-mm length, Varian Inc., Walnut Creek, CA, United States). The mixed solution of methanol:acetic acid:water (12:1:88, v/v) was used as the eluent solution at a flow rate of 1.0 mL/min at the operating temperature of 25°C. The concentration of $NH_{4}^{+}$ was determined through a colorimetric assay (Kemper, 1974).

### 3.6 Data analysis

The data shown in this study were obtained from at least three independent experiments and expressed as the mean values ± standard deviations (SDs). Analysis of variance by *t*-test or one-way ANOVA was conducted using SPSS version 22 (IBM Corp., Armonk, NY, United States). Differences were considered significant at *p* < 0.05.

## 4 Results

### 4.1 Lignocellulosic compositions of casava pulp

The chemical composition of cassava pulp used in this study was analyzed, and the major components were 49.0% ± 0.2% (w/w) starch, 25.4% ± 0.2% (w/w) cellulose, 6.1% ± 0.01% (w/w) hemicellulose, 5.8% ± 0.01% (w/w) lignin and 11.0% ± 0.03% (w/w) others (such as protein, fat, ash) with a moisture content of 78.7% ± 0.5% (w/w), as shown in Table 1. Therefore, starch and lignocellulosic fibrous material remaining in cassava pulp are abundant tropical agro-industrial biowaste that can be revalorized to valuable and green products through integrative biorefineries. In this study, the lignocellulosic compositions of cassava pulp may be different from those reported previously depending on the starch milling process, plant variety, growth conditions, soil conditions, and other environmental factors, such as pH, temperature, fertilizer, watering and climate (Sriroth et al., 2000; Rattanachomsri et al., 2009; Virunanon et al., 2013; Sudha et al., 2015).

**TABLE 1:** Lignocellulosic compositions of cassava pulp.

Lignocellulosic composition (%) of cassava pulp						Country	References
Starch	Cellulose	Hemicellulose	Lignin	Others	Moisture		
40.6 ± 0.2	40.4 ± 0.2	6.1 ± 1.0	2.0 ± 0.1	3.2 ± 0.3	78.7 ± 0.5	Thailand	This study
56.0	35.9	n/a	n/a	5.4	74.7	Thailand	Sriroth et al. (2000)
60.1 ± 0.1	15.6	4.6	2.8 ± 0.1	n/a	n/a	Thailand	Rattanachomsri et al. (2009)
59.4 ± 0.02	25.8 ± 0.2	3.5 ± 0.7	n/a	2.1 ± 0.03	4.1 ± 0.03	Thailand	Akaracharanya et al. (2011)
75.1	4.11	4.20	1.15	n/a	n/a	Thailand	Virunanon et al. (2013)
29.4	17.7	4.6	2.8	n/a	n/a	India	Sudha et al. (2015)
83.8	n/a	n/a	n/a	4.0	n/a	Thailand	Khanpanuek et al. (2022)

### 4.2 Optimal conditions for microwave-assisted cassava hydrolysis

Factors influencing dilute acid hydrolysis, including reactant concentration, liquid-to-solid ratio, watt power and irradiation time, were carefully investigated in this study. The −20 mesh particle size was applied for cassava pulp hydrolysis according to a previous report (Sukruansuwan and Napathorn, 2018).

#### 4.2.1 Effects of dry casava pulp content on the hydrolysis reaction

**FIGURE 1:**
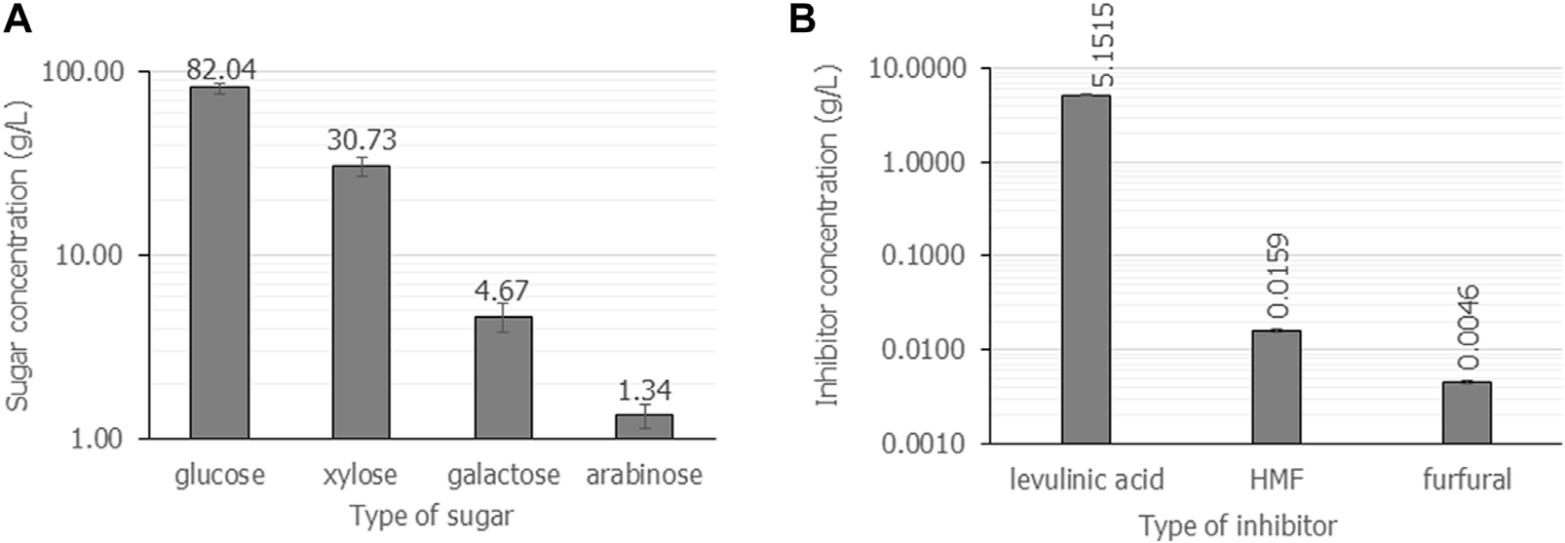
The composition of fermentable sugars **(A)** and inhibitors **(B)** when cassava pulp was hydrolyzed with liquid-to-solid ratios of 20 mL/g using 2.5% (v/v) H_2_SO_4_ under microwave irradiation at 800 W for 8 min. All the data are representative of the results of three independent experiments and are expressed as the mean values ± standard deviations (SD).

It has been reported the sugar yield from lignocellulosic materials increased as the liquid-to-solid ratio increased. Nevertheless, the overall cost of hydrolysis increases resulting in an increase in the cost of the subsequent fermentation and downstream processes (Chen, 2015). Generally, a liquid-to-solid ratio of 5 mL/g–20 mL/g was suggested for the acid hydrolysis reaction. In this study, the liquid-to-solid ratio was varied from 20 mL/g, 10 mL/g, 7.5 mL/g, and 5 mL/g (equal to the amount of dry casava pulp 5%, 10%, 15%, and 20% (w/v) in the 100 mL hydrolysis reaction). First, microwave irradiation was performed using 2.5% (v/v) H_2_SO_4_ at 800 W for 8 min. It was found that a liquid-to-solid ratio less than 20 mL/g was not suitable for the hydrolysis reaction, as starch granules absorbed all acid solution and became glutinous solution. Then, after microwave irradiation, the heat energy resulted in burnt and brown gelatinization (Supplementary Figure S1). The hydrolysate solution could not be separated from starch gelatinization. In addition, as shown in Figure 1, the liquid-to-solid ratio of 20 mL/g hydrolyzed with 2.5% (v/v) H_2_SO_4_, 800 W for 8 min gave the highest concentration of fermentable sugars and inhibitors. Notably, glucose (82.0 g/L ± 5.3 g/L) was a major component, followed by xylose (30.7 g/L ± 3.7 g/L), galactose (4.7 g/L ± 0.9 g/L) and arabinose (1.4 g/L ± 0.2 g/L). Fructose was not detected under this condition. The major inhibitor composition was levulinic acid (5.2 g/L ± 0.1 g/L), followed by 5-HMF (0.02 g/L ± 0.0 g/L) and furfural (0.005 g/L ± 0.0 g/L). The total concentration of fermentable sugar was 88.1 g/L ± 0.6 g/L, resulting in a yield coefficient of g-fermentable sugars from g-cassava pulp ( $\left.Y_{FS/CP}\right)$ of 0.88 g/g. The total amount of inhibitors was 5.17 g/L ± 0.03 g/L. Therefore, the optimal liquid-to-solid ratio was 20 mL/g, which was applied in all experiments.

#### 4.2.2 Effect of acid types and concentrations from casava pulp hydrolysis under microwave irradiation on the yield of sugars and inhibitors

Next, cassava pulp was hydrolyzed with three different solutions: H_3_PO_4_, H_2_SO_4_ and water. The concentration of acids was varied from 1%, 1.5%, 2%, 2.5%, and 3% (v/v) at different hydrolysis times for 1, 3, 6 and 8 min with an irradiation power of 800 W. The result shows that, as a control experiment, water hydrolysis of cassava pulp with a liquid-to-solid ratio of 20 mL/g at 800 W for 8 min gave 1.3 g/L ± 0.01 g/L, 1.9 g/L ± 0.01 g/L, 2.27 g/L ± 0.01 g/L, and 2.93 g/L ± 0.01 g/L of total fermentable sugars, respectively. The irradiation time had a positive effect on increasing fermentable sugar concentrations, which may be attributed to increasing the reactant temperature.

Consequently, the hydrolysis of casava pulp using a liquid-to-solid ratio of 20 mL/g by 1%, 1.5%, 2%, 2.5%, and 3.0% (v/v) H_3_PO_4_ at different irradiation times of 1, 3, 6 and 8 min was investigated. The irradiation power was set at 800 W. Unfortunately, H_3_PO_4_ gave a glutinous solution, and it was impossible to obtain filtrated hydrolysate, as shown in Supplementary Figure S2. Hence, the use of H_3_PO_4_ for acid hydrolysis was omitted in this study, as cassava pulp hydrolysis requires higher concentrations and stronger acids than H_3_PO_4_. The resulting fermentable sugars and inhibitors obtained from 3% H_3_PO_4_ at 800 W for 8 min are presented in Table 3.

**FIGURE 2:**
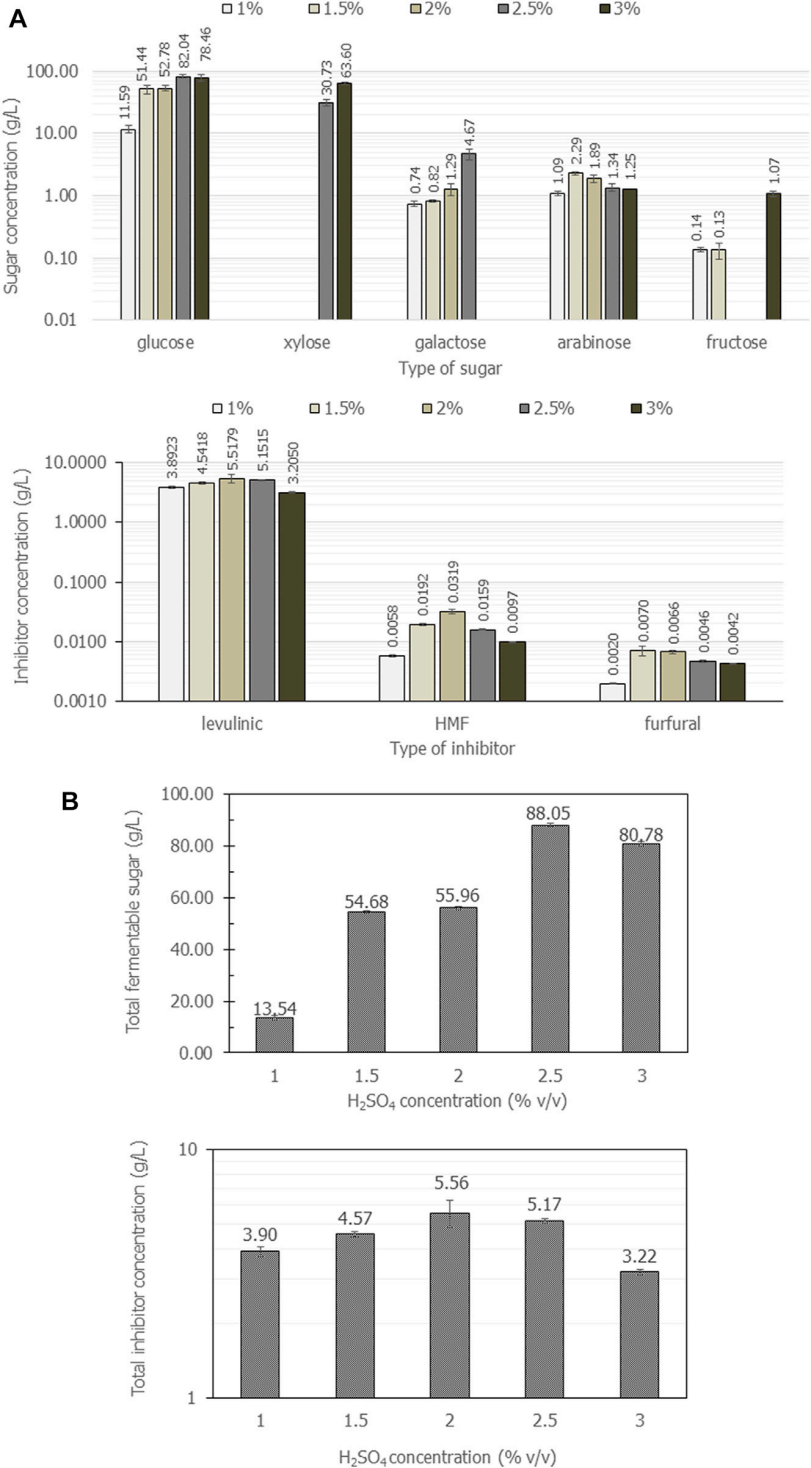
The composition of fermentable sugars and inhibitors **(A)** and total amount of fermentable sugars and inhibitors **(B)** when cassava pulp was hydrolyzed with liquid-to-solid ratios of 20 mL/g using 1%, 1.5%, 2%, 2.5%, and 4% (v/v) H_2_SO_4_ under microwave irradiation at 800 W for different times for 3, 5 and 8 min. All the data are representative of the results of three independent experiments and are expressed as the mean values ± standard deviations (SD).

In parallel, H_2_SO_4_ was tested under the same range of acid concentrations. Figure 2 shows the composition of fermentable sugars and inhibitors obtained from cassava pulp the hydrolysis with 1%, 1.5%, 2%, 2.5%, and 3% (v/v) H_2_SO_4_ under microwave irradiation at 800 W for 8 min. The results summarized in Figures 2A revealed that cassava pulp hydrolysis under microwave irradiation using 2.5% (v/v) H_2_SO_4_ gave the highest glucose concentration and total fermentable sugars. Notably, glucose was a major component in all experiments. Notwithstanding, at 3.0% (v/v) H_2_SO_4_, xylose was the major component (63.60 g/L ± 2.4 g/L), but total fermentable sugars decreased from 88.1 g/L ± 0.6 g/L to 80.8 g/L ± 0.7 g/L (Figure 2B). Xylose was not produced with H_2_SO_4_ concentrations lower than 2.0% (v/v) (Figure 2A). Additionally, the highest 5.6 g/L ± 0.69 g/L inhibitors were obtained with 2.0% (v/v) H_2_SO_4_ (Figure 2B), and levulinic acid was the major component. Overall, the concentration of 1.5% (v/v) (v/v) H_2_SO_4_ was the optimal acid concentration for cassava pulp hydrolysis under microwave irradiation based on unpresented xylose.

#### 4.2.3 Effect of microwave irradiation power and time on total fermentable sugars

**FIGURE 3:**
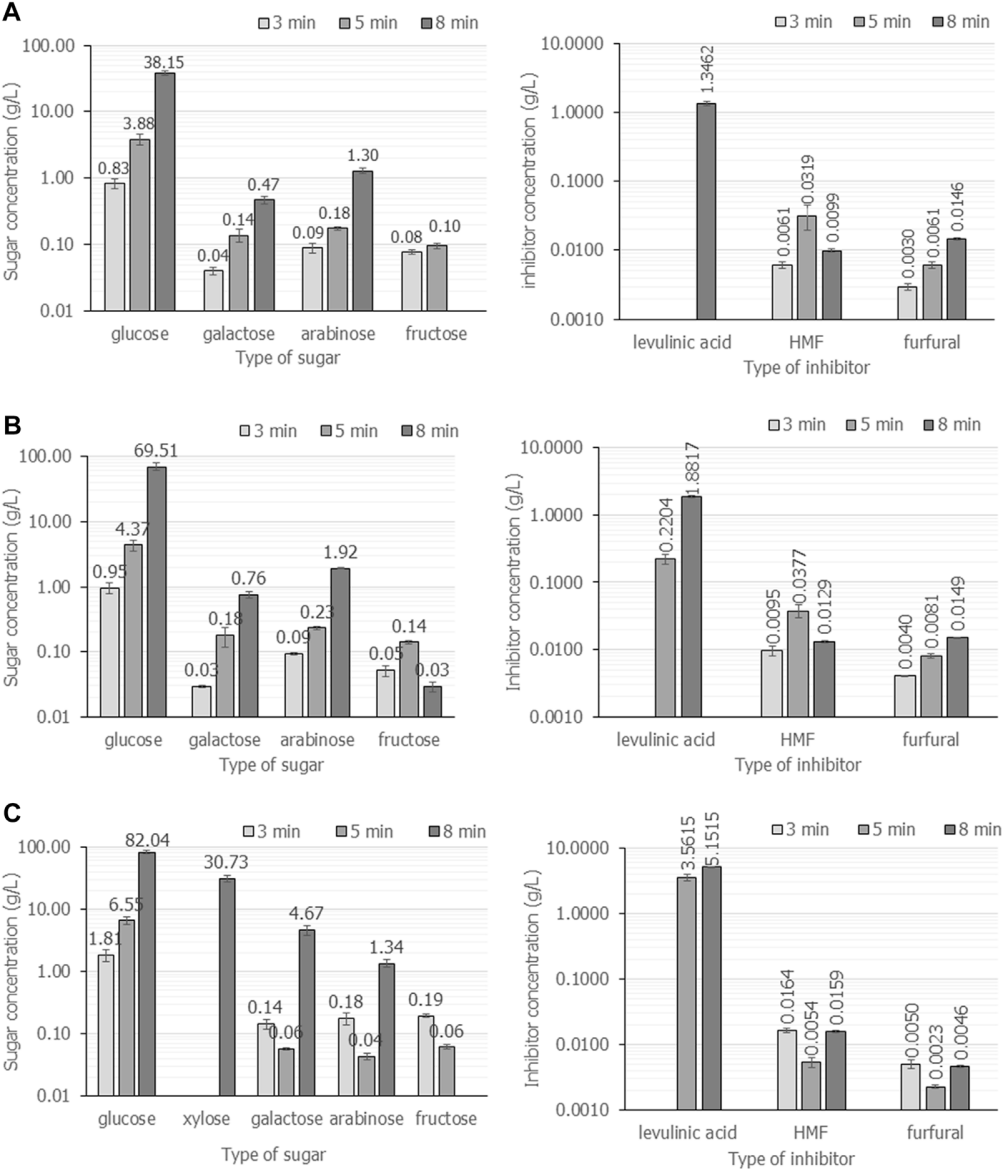
The composition of fermentable sugars and inhibitors when cassava pulp was hydrolyzed with liquid-to-solid ratios of 20 mL/g using 2.5% (v/v) H_2_SO_4_ under microwave irradiation at 600 W **(A)**, 700 W **(B)** and 800 W **(C)** for 3, 5 and 8 min, respectively. All the data are representative of the results of three independent experiments and are expressed as the mean values ± standard deviations (SD).

**FIGURE 4:**
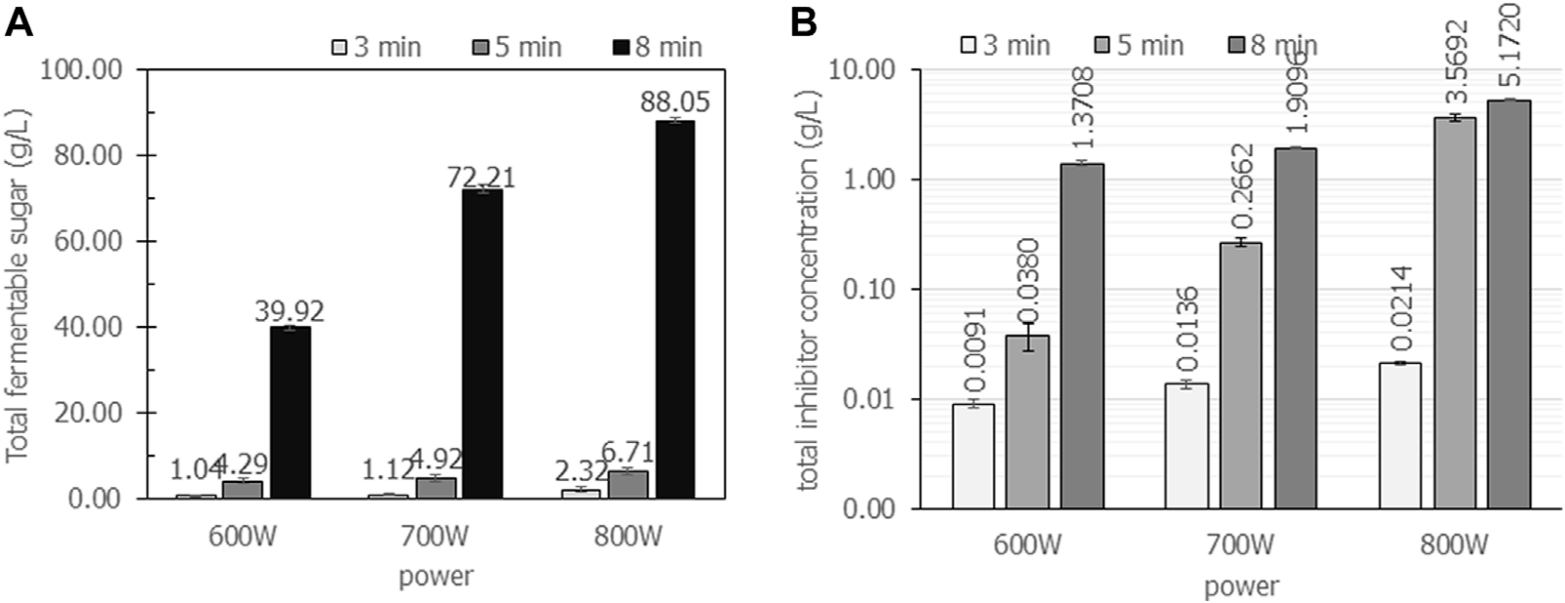
The total concentration of fermentable sugars and inhibitors when cassava pulp was hydrolyzed with liquid-to-solid ratios of 20 mL/g using 2.5% (v/v) H_2_SO_4_ under microwave irradiation at 600 W, 700 W and 800 W for 3, 5 and 8 min, respectively. All the data are representative of the results of three independent experiments and are expressed as the mean values ± standard deviations (SD).

The wattage power and time were carefully investigated in detail for cassava pulp hydrolysis using H_2_SO_4_. The summarized results are demonstrated in Figure 3; Table 2. In Figure 3, the cassava pulp hydrolysis condition was 2.5% (v/v) H_2_SO_4,_ and the irradiation power was varied at 600 W (Figure 3A), 700 W (Figure 3B) and 800 W (Figure 3C) for 3, 5 and 8 min, respectively. The total fermentable sugars increased from 39.0 g/L ± 0.6 g/L ( $Y_{FS/CP}$ = 0.39 g/g) to 72.2 ± 0.8 ( $Y_{FS/CP}$ = 0.72 g/g) and 88.1 g/L ± 0.7 g/L ( $Y_{FS/CP}$ = 0.88 g/g) as the irradiation power increased from 600 W to 800 W, respectively. The optimal irradiation power was 800 W for 8 min, resulting in the highest yields of $Y_{FS/CP}$ = 0.88 g/g and $Y_{S_{PHB}/CP}$ = 0.82 g/g. The major composition of the inhibitor was levulinic acid, followed by HMF and trace amounts of furfural. As displayed in Figure 4, the total inhibitor concentration in all case studies was lower than 5.2 g/L. The use of fermentable sugars at 88.1 g/L ± 0.7 g/L requires dilution in the range of 20 g/L–40 g/L so that inhibitors are also diluted. Thus, inhibitor removal prior to the use of hydrolysate was unnecessary.

**TABLE 2:** Effect of wattage power and time on sugar yield and reactant temperature. Five grams of samples were hydrolyzed with 2.5% (v/v) H_2_SO_4_ and wattage power was varied at 600 W, 700 W, and 800 W for 3, 5 and 8 min.

Time (min)	Temperature (°C)	Sugars (g/L)					Total FS (g/L)	$Y_{FS/CP}$ (g/g)	$S_{PHB}$ (g/L)	$Y_{S_{PHB}/CP}$ (g/g)
		Glucose	Fructose	Galactose	Arabinose	Xylose				
600 W										
3 min	56	0.8 ± 0.2	0.1 ± 0.1	0.04 ± 0.0	0.1 ± 0.0	-	1.0 ± 0.1	0.01	0.9 ± 0.1	0.01
5 min	96	3.9 ± 0.7	0.1 ± 0.0	0.1 ± 0.1	0.2 ± 0.0	-	4.3 ± 0.7	0.04	4.9 ± 0.7	0.04
8 min	100	38.2 ± 3.1	0.0 ± 0.0	0.5 ± 0.1	1.3 ± 0.1	-	39.9 ± 3.1	0.40	38.2 ± 3.1	0.38
700 W										
3 min	62	1.0 ± 0.2	0.1 ± 0.0	0.03 ± 0.1	0.1 ± 0.0	-	1.1 ± 0.1	0.01	1.1 ± 0.1	0.01
5 min	98	4.4 ± 0.8	0.1 ± 0.1	0.2 ± 0.1	0.2 ± 0.0	-	4.9 ± 0.8	0.05	4.5 ± 0.8	0.05
8 min	100	69.5 ± 9.1	0.03 ± 0.1	0.8 ± 0.1	1.9 ± 0.0	-	72.2 ± 9.1	0.72	69.5 ± 4.1	0.70
800 W										
3 min	74	1.8 ± 0.4	0.2 ± 0.1	0.1 ± 0.0	0.2 ± 0.0	-	2.3 ± 0.4	0.02	2.0 ± 0.1	0.02
5 min	99.5	6.6 ± 0.8	0.1 ± 0.1	0.1 ± 0.0	0.1 ± 0.0	-	6.7 ± 0.8	0.07	6.7 ± 0.8	0.06
8 min	100	82.0 ± 5.2	0.0 ± 0.0	4.7 ± 0.9	1.3 ± 0.2	30.7 ± 3.7	88.1 ± 0.7	0.88	82.0 ± 5.2	0.82

$S_{PHB}$ = PHB substrates (glucose and fructose).

The effect of irradiation power and time on the total concentration of sugars and inhibitors is clearly observed. The maximum power of the household microwave is 800 W. An irradiation time above 8 min results in boiling of the acid solution that causes volatile acids and is harmful to the operator. The effect of irradiation power and time on reactant temperature is depicted in Table 2. Increasing irradiation time resulted in increasing reaction temperature. For an irradiation time of 8 min, the reaction temperature reached 100°C in all experiments. In addition, the sugar concentration of 88.1 g/L ± 0.7 g/L containing glucose concentration of 82.0 g/L ± 5.2 g/L would be enough for further microbial conversion to value-added products, i.e., polyhydroxybutyrate production.

Finally, the effects of H_2_SO_4_ concentration, irradiation power and irradiation time on total fermentable sugars and inhibitors are summarized in Table 3. The fermentable sugars increased as the H_2_SO_4_ concentration, irradiation power and time increased. The hydrolysate obtained from 2.0% (v/v) or 1.5% (v/v) H_2_SO_4_ with an irradiation power of 800 W for 8 min was suitable for microorganisms sensitive to xylose.

**TABLE 3:** Effect of H_2_SO_4_ concentration, irradiation power and time on total fermentable sugar. Five grams of samples were hydrolyzed with 1, 1.5, 2, 2.5, 3% (v/v) H_2_SO_4_ and 3% (v/v) H_3_PO_4_ and wattage power was varied at 600 W, 700 W, and 800 W for 3, 5 and 8 min.

Types of acid	Acid concentration % (v/v)	Irradiation power (watts)	Total fermentable sugar (g/L)			Total inhibitors (g/L)		
			3 min	5 min	8 min	3 min	5 min	8 min
H_2_SO_4_	1.0	600	1.0 ± 0.6	0.9 ± 0.4	5.8 ± 0.4	0.002 ± 0.00	0.004 ± 0.00	0.005 ± 0.00
		700	1.2 ± 0.5	2.5 ± 0.4	5.2 ± 0.6	0.002 ± 0.00	0.010 ± 0.00	0.005 ± 0.00
		800	1.3 ± 0.4	5.9 ± 0.5	13.5 ± 1.7	0.006 ± 0.00	1.157 ± 0.00	3.900 ± 0.17
	1.5	600	0.7 ± 0.1	3.4 ± 0.2	24.4 ± 1.0	0.006 ± 0.00	0.015 ± 0.00	0.425 ± 0.09
		700	1.4 ± 0.4	3.3 ± 0.3	20.0 ± 0.4	0.006 ± 0.00	0.619 ± 0.00	0.275 ± 0.06
		800	1.3 ± 0.6	4.2 ± 0.4	54.7 ± 7.4	0.011 ± 0.00	2.549 ± 0.17	4.568 ± 0.12
	2.0	600	0.6 ± 0.1	2.6 ± 0.5	49.7 ± 0.4	0.004 ± 0.00	0.351 ± 0.00	1.734 ± 0.08
		700	1.5 ± 0.1	0.7 ± 0.4	40.9 ± 0.6	0.007 ± 0.00	0.283 ± 0.00	2.328 ± 0.12
		800	1.7 ± 0.8	11.6 ± 0.5	56.0 ± 4.7	0.011 ± 0.00	1.869 ± 0.04	5.557 ± 0.69
	2.5	600	1.0 ± 0.1	4.3 ± 0.7	39.9 ± 0.6	0.009 ± 0.00	0.038 ± 0.01	1.371 ± 0.07
		700	1.1 ± 0.1	4.9 ± 0.9	72.2 ± 0.8	0.014 ± 0.00	0.266 ± 0.02	1.909 ± 0.05
		800	2.3 ± 0.5	6.7 ± 0.8	88.1 ± 0.7	0.021 ± 0.00	3.569 ± 0.25	5.172 ± 0.10
	3.0	600	1.1 ± 0.6	3.1 ± 0.6	21.2 ± 0.3	0.41 ± 0.01	0.002 ± 0.00	1.120 ± 0.08
		700	12.5 ± 0.6	9.3 ± 0.6	63.0 ± 0.8	0.08 ± 0.01	0.387 ± 0.02	1.958 ± 0.07
		800	16.1 ± 0.2	14.9 ± 0.8	80.8 ± 1.7	0.09 ± 0.04	2.825 ± 0.17	3.881 ± 0.13
H_3_PO_4_								
	3.0	800	n.d	n.d	43.0 ± 1.7	n.d	n.d	0.792 ± 0.02

Overall, as shown in Table 2, the irradiation power and time play the most important role in the internal thermal effect which is generated by the direct interaction between the heated material and electromagnetic field in microwave heating, and consequently producing rapid and volumetric heating (Özbek et al., 2021). In addition to the thermal effects of microwave, the non-thermal effects also provide a physical explosion effect within the microfibers, which speed up the breakdown of the resistant lignocellulosic structure. These abovementioned factors may cause a shorter time in the cassava pulp conversion to sugars by the application of microwave with dilute acid catalyst.

### 4.3 PHB production by *Cupriavidus necator* strain A04 using cassava pulp hydrolysate without removal of inhibitors

**FIGURE 5:**
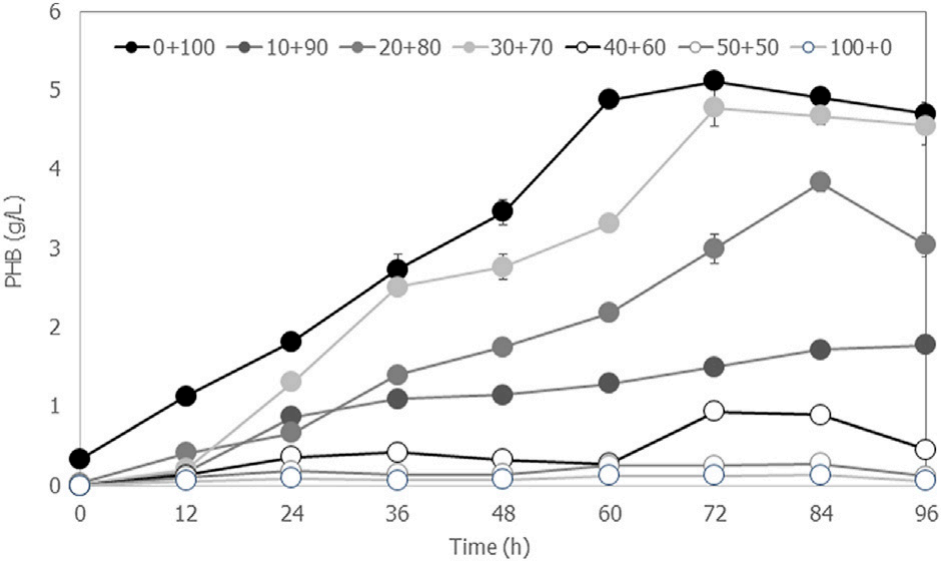
Time courses of PHB production by *Cupriavidus necator* strain A-04 when the hydrolysate was mixed with mineral salt medium at ratios of 0:100, 10:90, 20:80, 30:70, 40:60, 50:50 and 100:0 (v/v), where the C/N ratio was set at 200. All the data are representative of the results of three independent experiments and are expressed as the mean values ± standard deviations (SD).

The high concentration of fermentable sugars obtained from microwave-assisted acid cassava pulp hydrolysis can be considered a food waste biorefinery framework. Glucose was the major component containing low concentrations of inhibitors. Therefore, the hydrolysates can be applied in various aspects for microbial products without the need for alkaline pretreatment and inhibitor removal steps. In this study, a case study was tested for biodegradable polyhydroxybutyrate (PHB) production. In a previous study, *Cupriavidus neactor* strain A-04 was reported to tolerate inhibitors present in the hydrolysate prepared from pineapple core and pineapple peel and produced PHB without removal of inhibitors (Sukruansuwan and Napathorn, 2018). Thus, *C. necator* strain A-04 was chosen as a model for converting hydrolysate prepared from cassava pulp to PHB. The $NH_{4}^{+}$ concentration in the hydrolysate was determined to be only 0.06 g/L. The hydrolysate was mixed with mineral salt medium at a ratio of 0:100, 10:90, 20:80, 30:70, 40:60, 50:50 and 100:0 (v/v), where the C/N ratio was set at 200 and $S_{PHB}$ was adjusted to 30 g/L in all experiments. The control experiment was *C. necator* strain A-04 cultured in MSN medium consisting of 30 g/L glucose with a C/N ratio of 200. The time course of PHB production from each experiment is compared in Figure 5, and the kinetics of cell growth, the coefficient yield (g-PHB/g- $S_{PHB}$ ) and specific PHB production rate by *C. necator* strain A-04 are summarized in Table 4. It was found that hydrolysate alone did not support PHB production, although cells could grow on the hydrolysate alone. The optimal ratio between hydrolysate and MSN medium was 30:70 (v/v), which gave PHB concentrations similar to those obtained from the control experiment. Thus, this study proposed an easy hydrolysis method for the preparation of fermentable sugars within 8 min that is very attractive for industrial-scale production.

**TABLE 4:** Kinetics of cell growth, sugar consumption and PHB production by *Cupriavidus necator* strain A-04 using hydrolysate mixed with mineral salts medium 0:100, 10:90, 20:80, 30:70, 40:60, 50:50 and 100:0 (v/v) where C/N ratio was set at 200 in all experiments.

Kinetic parameters	0:100	10:90	20:80	30:70	40:60	50:50	100:0
PHB concentration (g/L)	5.1 ± 0.1	1.8 ± 0.0	3.8 ± 0.1	4.8 ± 0.2	0.9 ± 0.0	0.3 ± 0.0	0.1 ± 0.0
PHB content (%wt)	73.7 ± 0.6	28.3 ± 0.1	43.9 ± 0.2	66.8 ± 0.3	18.7 ± 0.1	5.0 ± 0.1	1.0 ± 0.0
Specific growth rate (1/h)	0.01	0.016	0.014	0.014	0.013	0.016	0.02
Specific production rate (g-PHB/g-CDW/h)	0.03	0.004	0.007	0.02	0.002	0.001	0.017
$Y_{P/S}$ (g-PHB/g- $S_{PHB}$ )	0.4	0.17	0.26	0.35	0.1	0.05	0.001

## 5 Discussion

To comply with a circular bioeconomy, the conversion of lignocellulosic agricultural and forestry wastes to biofuels, chemicals and biopolymers using clean and green processes has been considered a second-generation biorefinery. As demonstrated in Table 1, cassava pulp is a rich starch waste containing 40%–80% starch and 20%–40% fibers depending on the starch milling technology, plant variety and other environmental factors (Zhang et al., 2016; Bunterngsook et al., 2017). The milling process can be operated either manually or mechanically. For instance, the effect of starch milling machines on cooked cassava flour quality has been studied using the milling commenced with mortar mill, attrition, hammer, and the pin mills (Adesina and Bolaji, 2013). The composition of cassava pulp varied due to the difference in starch extraction efficiency which contributes to starch content remaining in fibers. The cassava pulp used in this study was obtained using the wet milling process with a saw-tooth rasper (Sriroth et al., 2000; Tran et al., 2015). In this study, the cassava pulp consisted of 40.6% ± 0.2% starch, 40.4% ± 0.2% cellulose and 6.1% ± 1.0% hemicellulose. Due to the effectiveness of the milling process utilized by the starch producing company, the starch concentration trapped in cassava pulp was one of the lowest in Thailand (see Table 1). The lignin content was only 2.0% ± 0.1%, so alkaline pretreatment to remove lignin was not necessary.

Lignocellulosic biomass typically has a lignin that is the most recalcitrant component and consists of three major components: cellulose, hemicellulose and lignin (Chuetor et al., 2021). Among the most abundant lignocellulosic biomass for biorefineries, cassava pulp has been one of the most studied crop wastes after rice and corn in various utilization options. The hydrolysis of cassava pulp has been reported based on various approaches, such as acid hydrolysis (Phowan and Danvirutai, 2014; Kurdi and Hansawasdi, 2015), enzymatic hydrolysis (Bunterngsook et al., 2017; Siriwong et al., 2019), and a combination of acid and enzymatic hydrolysis (Thongchul et al., 2010; Wattanagonniyom et al., 2017; Efeovbokhan et al., 2019; Areepak et al., 2022). Recently, microwave-assisted hydrolysis has gained some interest as it is an express and simple method for adopting biorefineries (Tsubaki et al., 2008; Hermiati et al., 2012; Klangpetch et al., 2022). In this study, household microwaves were chosen and tested for their potential use in microwave-assisted hydrolysis strategies due to their price and easy operation.

The highest fermentable sugar concentration of 88.05 g/L ± 7.65 g/L was obtained with 2.5% (v/v) H_2_SO_4,_ whereas 3%, 2%, 1.5%, and 1% (v/v) H_2_SO_4_ resulted in fermentable sugar concentrations of 80.78 g/L ± 10.72 g/L, 55.96 g/L ± 4.66 g/L, 54.68 g/L ± 7.41 g/L, and 13.54 g/L ± 1.67 g/L, respectively. The effect of acid concentration in this study was similar to previous reports that used microwave-assisted pretreatment of sweet sorghum bagasse and the obtained hydrolysate was used for bioethanol production. The concentration of H_2_SO_4_ was varied from 1%, 3%, 5%, and 7% (v/v) under microwave irradiation at 300 W for 10 min. The total sugar concentration increased when the acid concentration increased (Ndaba, 2013). It has been reported that acid hydrolysis techniques was classified to two types based on acid concentration: concentrated acid hydrolysis at a low temperature or diluted acid hydrolysis at a high temperature (Chen, 2015; Saadon et al., 2022). Concentrated acid hydrolysis, 72% H_2_SO_4_, 42% HCl or 83% H_3_PO_4_ phosphoric acid, can completely solubilize crystalline cellulose at low temperatures of approximately 25°C–70°C, resulting in the homogeneous hydrolysis of cellulose. Diluted acid hydrolysis is normally operated with concentrations of 0.3%–2.5% at high temperatures of approximately 120°C–210°C (Rashid et al., 2021). In comparison, concentrated acid hydrolysis facilitate effective lignocellulosic hydrolysis better than dilute acid hydrolysis because two steps hydrolysis were simultaneously occurred: a decrystallization step that the crystal structure of lignin and fiber were swollen and digested by more than 60% (v/v) H_2_SO_4_ and a hydrolysis step with 20%–30% (v/v) H_2_SO_4_ to deliver sugars from the decrystallized fiber (Zhang and Bao, 2020). For instance, mixed wood chips hydrolysis using concentrated acid yielded 72%–82% glucose (Iranmahboob et al., 2002). The inhibitors as byproducts of microwave-assisted cassava pulp hydrolysis were very low due to the use of diluted acid in a short heating time. In addition, this method does not require an alkaline pretreatment as well as inhibitor removal steps. The overall cost can be reduced from the reduction of waste treatment costs and the water used for washing and neutralizing (Cheng et al., 2010; Sukruansuwan and Napathorn, 2018).

The advantage of using acid hydrolysis is concurrent lignin extraction with simultaneous saccharification. In this study, with the employment of microwave irradiation-assisted acid hydrolysis, lignin was not degraded to microbial inhibitors due to diluted acid concentration and a short heating time, but the condition was favorable for converting hemicellulose, which is more susceptible to acid hydrolysis, into soluble sugars and oligomers (Samuel et al., 2010), and then cellulose and lignin because they possess the highest level of resist to acids hydrolysis. Generally, diluted acid hydrolysis is performed at a concentration of 10%. With the use of the microwave synthesis reactor, the operating temperature occurred in acid hydrolysis reaction can be modulated between 100°C and 240°C. The pressure can be raised up to more than 1 MPa, which is higher than the saturated vapor pressure of the liquid. The advantages of dilute acid hydrolysis include the use of low acid concentration that offers low impact on the environment and simple waste management, and low material costs. Nevertheless, the progress of the dilute acid hydrolysis development was very slow. The key issue is that the temperature is an important factor influencing the rate of hydrolysis. On one hand, the hydrolysis rate will increase by 1 time as the temperature rises by 10°C. On the other hand, high temperature will stimulate the rate of monosaccharide decomposition. In this regard, as acid hydrolysis under high temperature is applied, the reaction time should be shortened. The increases in acid concentration, therefore, led to the increase of overall processing costs. Additionally, the equipment also needs to be the corrosion-resistance materials, resulting in the increase of equipment costs, which hampers industrial production opportunity. Normally, the concentration of dilute acid should not more than 10%. The temperature of the reactant after microwave-assisted dilute acid hydrolysis is summarized in Table 2.

Prior literature on acid hydrolysis mostly emphasized inorganic acids, including HCl and H_2_SO_4_. The main reason is that acid hydrolysis with HCl is superior to that with H_2_SO_4_; however, waste treatment with HCl is more difficult than with H_2_SO_4_. Additionally, the process using HCl is more expensive than that using H_2_SO_4_ because it requires equipment that has the higher level of corrosion resistance. Sugars present in the hydrolysates (sucrose, glucose, fructose, galactose, arabinose, xylose) were considered fermentable sugars (sucrose, glucose, fructose, galactose, arabinose) and PHB substrates (glucose and fructose) (Chanprateep et al., 2008; Sukruansuwan and Napathorn, 2018). The organic compounds that were considered microbial growth inhibitors [levulinic acid (LA), 5-(hydroxymethyl)furfural (5-HMF) and furfural (FAL)] in the hydrolysate samples were also analyzed. Under this condition, the obtained total fermentable sugars were 88.1 g/L ± 0.7 g/L (82.0 g/L ± 5.2 g/L glucose, 4.7 g/L ± 0.9 g/L galactose and 1.3 g/L ± 0.3 g/L arabinose) and 30.7 g/L ± 3.7 g/L xylose. In addition, microbial growth inhibitors in the microwave-assisted acid hydrolysis were 5.15 g/L ± 0.13 g/L levulinic acid (LA), 0.01 ± 0.01 5-(hydroxymethyl) furfural (5-HMF) and 0.004 g/L ± 0.01 g/L furfural (FAL), respectively. Fermentable sugars yielded from g-dry pulp was 0.88 g/g-dry cassava pulp, which was similar to the previous report using acid hydrolysis with HCl under steam conditions at 121°C and 15 psi for up to 2 h (Thongchul et al., 2010). Based on the above results, microwave-assisted cassava pulp hydrolysis showed high potential for the preparation of low-cost fermentable sugars suitable for microbial conversion. There have been some reports on similar research rationals using microwave-assisted hydrothermal conditions. However, the microwave used in these studies was different from this study. Hermiati et al. (2012) applied a microwave reactor instrument with variable and controlled frequencies of microwave energy so that it could offer temperature control in the temperature range of 180°C–240°C. The hydrolysis was performed without the use of acids but with the combination of adding activated carbon to remove inhibitors during microwave-assisted hydrolysis. Their study demonstrated that the presence of 1.0 g/g activated carbon in microwave-assisted hydrolysis resulted in the highest yield of glucose from cassava pulp (52.27%) when the temperature was set at 210°C for 15 min. Nevertheless, the method and conditions proposed in this study offer a shorter time, lower temperature and easier procedure than those in this report.

The application of crude hydrolysate was demonstrated by culturing *Cupriavidus nector* strain A-04 for biodegradable PHB production. The PHB content was 66.8% ± 0.3% with 4.8 g/L ± 0.2 g/L PHB and 7.5 g/L ± 0.1 g/L CDM. The coefficient yield $Y_{P/S}$ (g-PHB/g- $S_{PHB}$ ) was 0.35 g/g, where those from MS medium containing 30 g/L glucose were 0.4 g/g and 73.7% ± 0.6% PHB content. These obtained results were higher than PHB production by *Bacillus megaterium* Ti3 using pretreated corn husk hydrolysate that produced 1 g/L PHB with 57.8% PHB content in 48 h (De Souza et al., 2020). Meanwhile, PHB production from cassava starch hydrolysate by *Cupriavidus* sp. KKU38 under N-limited conditions gave 5.97 g/L PHB with 61.60% PHB content which were slightly lower than those in this study (Poomipuk et al., 2014) (see Table 5 for the comparison of cassava pulp hydrolysis methods). The thermal and mechanical properties including its potential applications of PHB produced by *C. necator* strain A-04 has already been reported (Chanprateep and Kulpreecha, 2006; Wongmoon and Napathorn, 2022; Sinsukudomchai et al., 2023). Therefore, this study proposed an alternative process to convert cassava pulp waste to value-added bioproducts within biorefinery scenarios.

**TABLE 5:** Comparison of cassava pulp hydrolysis methods for preparation of fermentable sugars.

Industrial wastes	Amount	Method	Time	Sugar concentration	Final product	References
Rice straw	1 g	microwave irradiation at 680 W	24 min	sugar 75 g/L	sugar	Ma et al. (2009)
Cassava pulp	1 g	1 g/g activated carbon Microwave 210°C, pH 3.0	12 min	glucose 52.27%	sugar	Hermiati et al. (2012)
Cassava pulp	67 g/L	Enzymatic hydrolysis pH 4.5, 50°C	24 h	glucose 0.51 g/g	Ethanol	Virunanon et al. (2013)
Cassava starch	30 g	Enzymatic hydrolysis at 90°C, pH 6.5	45 min	159.06 g/L glucose	PHB	Poomipuk et al. (2014)
Cassava pulp	1 g	1.5%NaOH 90°C + α-amylase	30 min	reducing sugar 0.72 g	Ethanol	Sudha et al. (2015)
Sugarcane bagasse	0.2 g	0.2 M H_2_SO_4_% under microwave irradiation at 320 W and alkaline pretreatment	7 min	sugar 86%	sugar	Zhu et al. (2016)
Cassava pulp	100 g	1N HCl hydrolysis 121°C	15 min	reducing sugar 31.6 g/L (0.42 g/g)	Ethanol	Wattanagonniyom et al. (2017)
		cellulase, α-amylase	28 h	reducing sugar 34.9 g/L (0.28 g/g)		
Pineapple core	10 g	1.5% (v/v) H_2_SO_4_ Autoclave 121°C	15 min	fermentable sugar 0.81 g/g	PHB	Sukruansuwan and Napathorn (2018)
Pineapple peel	10 g	1.5% (v/v) H_3_PO_4_ Autoclave 121°C	15 min	fermentable sugar 0.69 g/g	PHB	Sukruansuwan and Napathorn (2018)
Maple leaves and fresh grass	300 mg	0.2 M H_2_SO_4_% under microwave irradiation at 150 W	4 h	glucose 55.9%	sugar	Jiang et al. (2019)
Cotton towel	100 g	freeze-thawing with 3% NaOH, microwave treatment 200°C, 2% sulfuric acid	30 s	glucose 40.5 g	sugar	Sasaki et al. (2021)
Wheat straw	100 g	freeze-thawing with 3% NaOH, microwave treatment 200°C, 0.5% sulfuric acid	3 min	glucose 14.7 g	sugar	Sasaki et al. (2021)
Pistachio shell	1 g	1.96 N NaOH, microwave irradiation of 224 W, 2.63 min + enzymatic hydrolysis	72 h	glucose 82.67 mol%	sugar	Özbek et al. (2021)
Cassava pulp	1 g	2.5% (v/v) H_2_SO_4_ under microwave irradiation at 800 W	8 min	fermentable sugars 88.1 ± 0.7 g/L (0.88 g/g)	PHB	This study

## 6 Conclusion

This study demonstrated the factors affecting fermentable sugar and inhibitor concentrations generated by microwave-assisted hydrolysis of cassava pulp as a food waste biorefinery for PHB production. The results are summarized in Table 3. According to this study, the optimal liquid-to-solid ratio was 20 mL/g. Hydrolysis using H_3_PO_4_ was not possible for microwave-assisted cassava pulp hydrolysis, which could require higher acid concentrations that will increase the overall cost. The conditions proposed in this study offer a short irradiation time of 8 min with 800 W for all concentrations of H_2_SO_4_. At 2.5% (v/v) H_2_SO_4_ with an irradiation power of 800 W for 8 min, a nearly 82% yield of glucose and 88% yield of fermentable sugars were obtained from untreated cassava pulp. However, xylose was also generated under this intense condition, where a concentration of H_2_SO_4_ lower than 2.5% (v/v) did not generate xylose. Because levulenic acid is generated by acid-catalyzed dehydration of glucose, therefore, under 3.0% (v/v) H_2_SO_4_ with an irradiation power of 800 W for 8 min, the glucose concentration was lower than that obtained from 2.5% (v/v) H_2_SO_4_. Additionally, inhibitor concentrations were higher than those obtained from acid concentrations below 2.5% (v/v). This study suggested that diluted acid concentrations were more effective than concentrated acid concentrations. This simple chemical process, which requires neither food/feed-based alternatives nor cellulase enzymes, could enable crude fermentable sugars to be the renewable carbon source of microorganisms for a scalable biorefinery. *C. nector* strain A-04 could utilize fermentable sugars from crude hydrolysate. A 66.8% ± 0.3% PHB content with 4.8 g/L ± 0.2 g/L PHB and 7.5 g/L ± 0.1 g/L CDM was obtained with a coefficient yield $Y_{P/S}$ (g-PHB/g- $S_{PHB}$ ) of 0.35 g/g.

## Data Availability

The raw data supporting the conclusion of this article will be made available by the authors, without undue reservation.
