# Supplementary figures and images for: Microwave-assisted cassava pulp hydrolysis as food waste biorefinery for biodegradable polyhydroxybutyrate production
Source: Front Bioeng Biotechnol. 2023 Mar 6;11:1131053. doi: 10.3389/fbioe.2023.1131053 (PMC10025311; doi:10.3389/fbioe.2023.1131053)

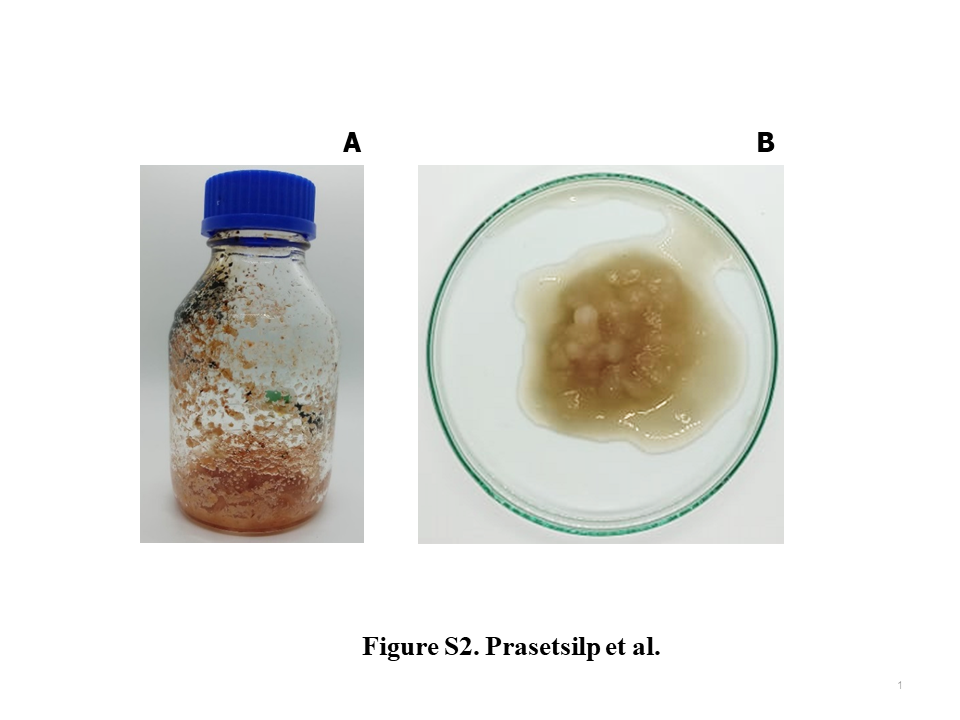

Supplement: Supplementary file 1 [file Image2.tif]

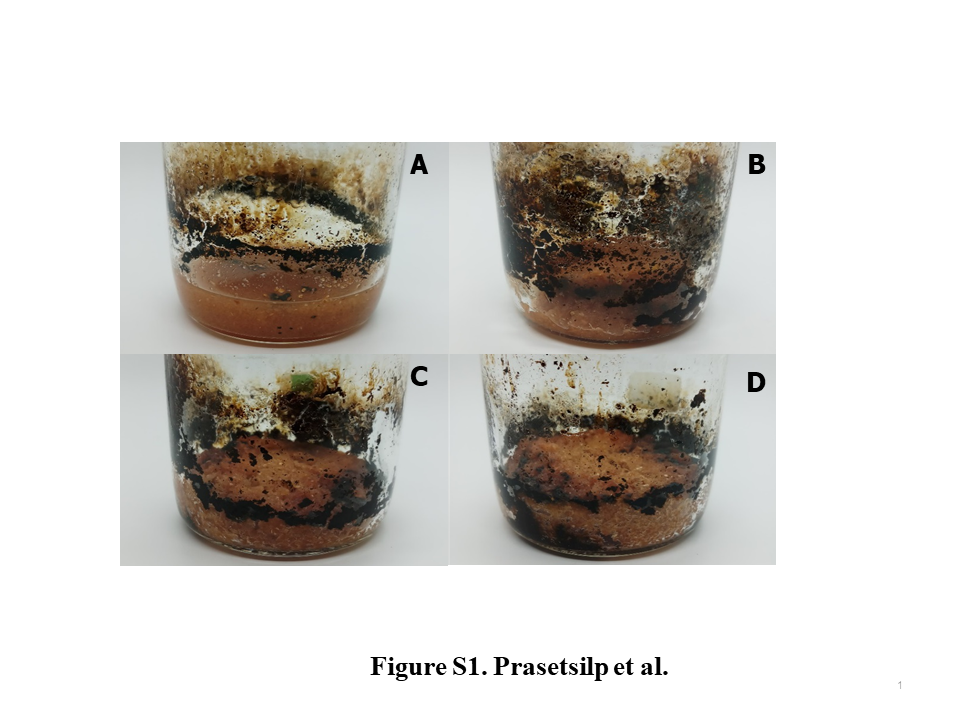

Supplement: Supplementary file 2 [file Image1.tif]
